# Anti-apoptotic gene transcription signature of salivary gland neoplasms

**DOI:** 10.1186/1471-2407-12-61

**Published:** 2012-02-07

**Authors:** Carolina Cavaliéri Gomes, Vanessa Fátima Bernardes, Marina Gonçalves Diniz, Luiz De Marco, Ricardo Santiago Gomez

**Affiliations:** 1 Department of Pathology, Universidade Federal de Minas Gerais, Belo Horizonte, Brazil; 2 Department of Oral Surgery and Pathology, Universidade Federal de Minas Gerais, Belo Horizonte, Brazil; 3 Department of Surgery, Universidade Federal de Minas Gerais, Belo Horizonte, Brazil

**Keywords:** Salivary gland neoplasms, Pleomorphic adenoma, Apoptosis, Bcl-2, Bax, Caspase 3, Transcription, p53, Cell proliferation, Tumour size

## Abstract

**Background:**

Development of accurate therapeutic approaches to salivary gland neoplasms depends on better understanding of their molecular pathogenesis. Tumour growth is regulated by the balance between proliferation and apoptosis. Few studies have investigated apoptosis in salivary tumours relying almost exclusively on immunohistochemistry or TUNEL assay. Furthermore, there is no information regarding the mRNA expression profile of apoptotic genes in salivary tumors. Our objective was to investigate the quantitative expression of *BCL-2 *(anti-apoptotic), *BAX *and *Caspase3 *(pro-apoptotic genes) mRNAs in salivary gland neoplasms and examine the association of these data with tumour size, proliferative activity and p53 staining (parameters associated with a poor prognosis of salivary tumours patients).

**Methods:**

We investigated the apoptotic profile of salivary neoplasms in twenty fresh samples of benign and seven samples of malignant salivary neoplasms, using quantitative real time PCR. We further assessed p53 and ki-67 immunopositivity and obtained clinical tumour size data.

**Results:**

We demonstrated that *BCL-2 *mRNA is overexpressed in salivary neoplasms, leading to an overall anti-apoptotic profile. We also found an association between the anti-apoptotic index (*BCL-2/BAX*) with p53 immunoexpression. A higher proliferative activity was found in the malignant tumours. In addition, tumour size was associated with cell proliferation but not with the transcription of apoptotic genes.

**Conclusion:**

In conclusion, we show an anti-apoptotic gene expression profile in salivary neoplasms in association with p53 staining, but independent of cell proliferation and tumour size.

## Background

Salivary gland tumours have an annual global incidence between 0.4 and 13.5 cases per 100 000 individuals [1]. High proliferative activity, presence of residual tumour and advanced tumour stage were shown to be strong negative predictors of survival in salivary gland neoplasms [2]. Development of targeted therapy calls for a better understanding of their molecular and cellular biology [3].

Apoptosis is a highly regulated active process, characterized by cell shrinkage, chromatin condensation and DNA fragmentation promoted by endonucleases. Induction of apoptosis is a normal defense against loss of growth control which follows DNA mutations. Apoptosis is frequently deregulated in human cancers, being a suitable target for anticancer therapy [4].

The B-cell lymphoma (BCL-2) family comprises different regulators involved in apoptosis. *BCL-2 *is an important proto-oncogene located at chromosome 18q21 [5]. It was the first gene implicated in the regulation of apoptosis. Its protein is able to stop programmed cell death (apoptosis) facilitating cell survival independent of promoting cell division [6]. *BCL-2 *is thought to be involved in resistance to conventional cancer treatment and its increased expression has been implicated in a number of cancers [4]. Apparently, many cancers depend on the anti-apoptotic activity of *BCL-2 *for tumour initiation and maintenance [7].

*BAX *(Bcl-2-associated protein X) is the most characteristic death-promoting member of the BCL-2 family. The translocation of Bax protein from the cytosol to the mitochondria triggers the activation of the caspases cascade, leading to death [8]. Caspase 3 *(CASP3) *was first described in 1995 and once activated, is considered to be responsible for the actual demolition of the cell during apoptosis [9,10].

In salivary neoplasms, apoptosis has been investigated almost exclusively by means of immunohistochemistry. Bcl-2 and Bax proteins are expressed in most of the salivary gland neoplasms investigated, but Bcl-2 positivity was found in a lower percentage of mucoepidermoid carcinomas [11-15]. In the studies with TUNEL in salivary gland neoplasms, apoptotic activity was inversely associated with Bcl-2 immunoexpression [11,15]. In salivary gland carcinomas, TUNEL was associated with a poor prognosis, being correlated with p53 and ki-67 staining [16]. As the transcription of apoptosis related genes could help to elucidate the pathogenesis of tumours, we propose to investigate the quantitative expression of *BCL-2 *(anti-apoptotic), *BAX *and *Caspase3 *(pro-apoptotic genes) using qPCR. As tumour size, high proliferative activity and p53 staining are associated with a poor prognosis of salivary tumours patients [2,17], we tested the association of these parameters with the transcription of the apoptotic/anti-apoptotic genes.

## Methods

### Samples

Twenty seven salivary gland neoplasms were included in this study. Fresh tumour samples were obtained from patients who underwent surgical excision of salivary gland neoplasms. The study was approved by the local ethics committee. The diagnoses were reviewed and confirmed: 17 pleomorphic adenomas, one basal cell adenoma, one Warthin tumour, one mucinous cystadenoma, three polymorphous low grade adenocarcinomas, two low grade mucoepidermoid carcinomas, one adenoid cystic carcinoma and one cystadenocarcinoma. Six samples of normal salivary glands obtained from healthy volunteers undergoing surgery for non-neoplastic disease were used as controls.

For each sample, a portion of the lesion was stored in RNAHolder (BioAgency Biotecnologia, São Paulo, SP, Brazil) at -80°C, while another portion was fixed in 10% buffered formalin and paraffin embedded. All the samples underwent the same fixation and processing procedures.

### Quantitative reverse transcriptase PCR (qRT-PCR)

Total RNA was isolated from samples using Tri-Phasis Reagent (BioAgency, São Paulo, Brazil) and treated with DNase (Invitrogen Life Technologies, Carlsbad, CA, USA). cDNA was synthesized with Superscript First-Strand Synthesis System kit (Invitrogen Life Technologies, Carlsbad, CA, USA) according to the manufacturer's instructions. Quantitative PCR analyses were carried out using 1x SYBR Green PCR Master Mix (Applied Biosystems, Warrington, CHS, UK). *BCL-2, BAX*, *CASP3 *and *ACTB *primers were designed using Primer Express software (Applied Biosystems, Foster City, CA, USA) version 3.0. The primer sequences are specified in Table 1. Reactions were performed in duplicate and run on a Step One machine (Applied Biosystems, Foster City, CA, USA). The cycling parameters were 10 min denaturation at 95°C followed by 40 cycles at 95°C for 15 s and 56°C for 1 min. The cycling was followed by melting curve analysis to distinguish specificity of the PCR products. *BCL-2*, *BAX *and *CASP3 *expressions were normalized with actin (*ACTB) *as internal control. The average threshold cycle (Ct) for two replicates per sample was used to calculate ΔCt. Relative quantification of these genes expressions was calculated with the 2^-ΔΔCt ^method. Normal salivary gland samples were used as a calibrator.

**Table 1:** qRT-PCR primer sequences and amplicon sizes

cDNA	Forward primer	Reverse primer	Product length
*BAX*	5'GAGCTGCAGAGGATG ATT GC 3'	5'CAGCTGCCACTCGGAAAA3'	70 bp

*BCL-2*	5'AGGCTGGGATGCCTTTGT3'	5'CAGCCAGGAGAAATCAAACAG3'	67 bp

*CASP3*	5'TCATAAAAGCACTGGAATGACATC3'	5'TTCTGAATGTTTCCCTGAGGTT3'	74 bp

*ACTB*	5'TGCCGACAGGATGCAGAAG3'	5'CTCAGGAGGAGCAATGATCTTGA3'	77 bp

Apoptosis tendency was estimated by two indexes: Anti-apoptotic index 1 (AI-1), calculated by the ratio between *BCL-2/BAX *expression and anti-apoptotic index 2 (AI-2), calculated by the ratio between *BCL-2/CASP*3. Samples with AI-1 or AI-2 higher than 1 were regarded as having higher anti-apoptotic activity than samples exhibiting an AI lower than 1.

### Immunohistochemistry

Paraffin-embedded sections (4 μm) were dewaxed in xylene, hydrated with graded ethanol and endogenous peroxidase blocked in 1% hydrogen peroxidase for 15 min. Antigen retrieval was performed with citric acid, pH 6.0. The primary antibodies used were ki-67 (MIB-1) and p53 (DO7), both from DAKO (Carpinteria, CA, USA) and diluted 1:50. Primary antiserum incubation was performed for 30 min at room temperature and binding visualized using a polymer-based system (EnVision, Dako Corporation, Carpinteria, CA, USA) with diaminobenzidine (Sigma, St Louis, MO, USA) as chromogen. For each antibody, positive (squamous cell carcinoma with known reactivity) and negative controls in which the primary antibody was omitted were included. The sections were counterstained with hematoxylin, dehydrated and mounted.

The percentage of ki-67 positive nuclei was obtained by counting nuclear staining in 10 high power fields (400 × magnification) including the most positive areas. Neoplasms were divided into two groups with 5% or more positive nuclei being considered as high proliferative activity, or fewer than 5% positive nuclei considered low proliferative activity. p53 stained nuclei were counted in eight fields (400 × magnification); more than 5% of positive nuclei was considered positive [18].

### Statistical Analyses

Mann-Whitney, Fisher Test and Spearman correlation tests were used when appropriate. P values < 0.05 were considered statistically significant. These tests were performed with BioEstat software (Belém, PA, Brazil), version 4.

## Results

### BAX, BCL-2 and CASP3 expression

There was no difference between mRNA expression of *BAX*, *BCL-2 *and *CASP3 *between malignant and benign salivary gland neoplasm groups. However, positive correlation was found using the Spearman test for all the following paired groups: *BAX/BCL-2 *(*p *= 0.010), *BAX/CASP3 *(*p *= 0.008), *BCL-2/CASP3 *(*p *< 0.0001). Overall, 78% (21/27) of the salivary neoplasm samples exhibited overexpression of *BCL-2 *mRNA compared to the expression in normal salivary glands, including 15 out of 17 pleomorphic adenomas. The expression of *BAX *and *CASP3 *in the salivary tumours, however, was higher than normal glands only in 52% (14/27) and 44% (12/27) samples, respectively.

### Anti-apoptotic indexes

**Figure 1:**
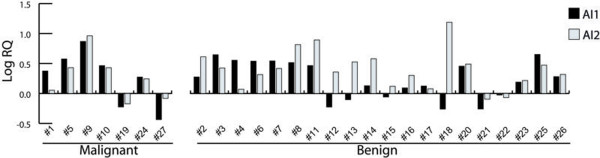
**Anti-apoptotic index 1 (black bars) and 2 (white bars) in the malignant and benign salivary gland neoplasms**. Most of the samples exhibited an anti-apoptotic profile. All the pleomorphic adenoma samples exhibited more *BCL-2 *than *CASP3 *transcription. Samples #19, #21 and #22, which showed a decreased AI-1 and 2, were p53 negative. X-axis represents the normal salivary glands pool of samples included as reference sample in all the reactions. AI-1 = *BCL-2/BAX*, AI-2 = *BCL-2/CASP*3.

**Figure 2:**
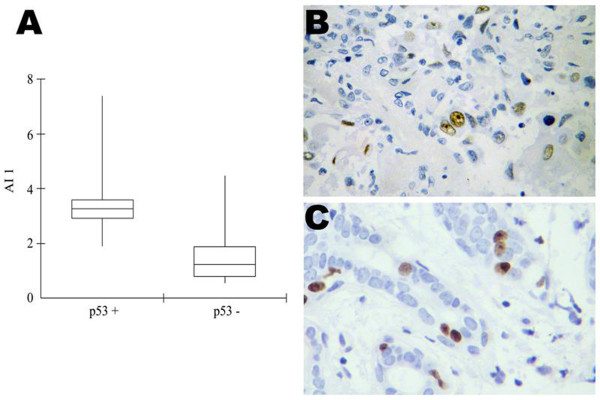
**Parameters statistically associated with p53 positivity**. (A) p53 immunopositivity was associated with a statistically significant higher anti-apoptotic index 1 (*p *= 0.004). (B) p53 immunostaining in a sample of pleomorphic adenoma (C) high proliferation index in a sample of polymorphous low grade adenocarcinoma. High proliferative activity was associated with p53 positivity. AI-1 = *BCL-2/BAX *(Original magnification 400×).

Anti-apoptotic index results are displayed in Table 2 and illustrated in Figure 1. Eighty-five percent (n = 23) of all salivary neoplasms samples showed a higher AI-1 or AI-2, when compared with normal salivary glands (Figure 1). AI-1 and AI-2 did not show association with malignancy. p53 immunopositivity was associated with a statistically significant higher AI-1 (*p *= 0.004) (Figure 2). AI-2 was not associated with any of the investigated parameters.

**Table 2:** Benign and malignant tumours data regarding diagnosis, tumour size, p53 staining, cell proliferation index and anti-apoptotic indexes 1 and 2

Sample	Diagnosis	Tumour size^a^	p53 immuno-staining	Cell proliferation	AI-1 (*BCL-2/BAX*)	AI-2 (*BCL-2/CASP3*)
#1	PLGA	2	+	high	2.370	1.132
#2	PA	1	+	low	1.887	4.092
#3	PA	1	-	low	4.423	2.665
#4	PA	2	+	high	3.584	1.177
#5	PLGA	2	+	high	3.765	2.689
#6	PA	2	+	high	3.461	2.066
#7	PA	-	-	low	3.493	2.613
#8	PA	2	+	low	3.274	6.520
#9	ACC	2	+	high	7.386	9.161
#10	PLGA	2	+	high	2.913	2.692
#11	PA	1	+	low	2.922	7.813
#12	PA	1	-	low	0.594	2.276
#13	PA	1	-	low	0.788	3.355
#14	PA	-	-	low	1.346	3.800
#15	PA	1	-	low	0.873	1.316
#16	PA	2	-	low	1.237	2.001
#17	MC	-	-	low	1.333	1.198
#18	PA	2	-	low	0.548	15.418
#19	CA	2	-	high	0.596	0.673
#20	PA	-	-	low	2.855	3.091
#21	WT	-	-	low	0.550	0.802
#22	BCA	-	-	low	0.943	0.854
#23	PA	-	-	low	1.548	1.646
#24	MEC	-	-	low	1.880	1.752
#25	PA	1	-	low	4.477	2.972
#26	PA	2	-	low	1.911	2.080
#27	MEC	1	not done	not done	0.366	0.828

^a ^Tumour size 1 refers to tumours ≤ 2 cm and tumour size 2 are tumours > 2 cm. PA = pleomorphic adenoma, MC = mucinous cystadenoma, WT = Warthin tumour, BCA = basal cell adenoma, PLGA = polymorphous low grade adenocarcinoma, ACC = adenoid cystic carcinoma, CA = cystadenocarcinoma, MEC = mucoepidermoid carcinoma. #27 paraffin embedded tissue was small and was not used in immunohistochemistry

### p53 and cell proliferation index

p53 and cell proliferation results are displayed in Table 2. The samples that were p53 positive in the immunohistochemistry also showed a high relative quantification of *BCL-2 *(*p *= 0.0003) and *CASP3 *mRNA (*p *= 0.0007). p53 positivity was also associated with high cellular proliferation index (*p *= 0.002) (Figure 2). In addition, the samples exhibiting high cellular proliferation index were associated with high *CASP3 *transcriptional levels (*p *= 0.018). High proliferation index was associated with malignancy (*p *= 0.001), as well.

### Tumour size

Samples were divided into two groups: tumour size ≤ 2 cm and tumour size > 2 cm. Tumour size showed association with a high cellular proliferation index (*p *= 0.019), despite showing no association with the transcription of *BCL-2, BAX *or *CASP3*, or with the anti-apoptotic indexes.

## Discussion

Very little is known about the apoptotic index of salivary gland neoplasms. We used two different anti-apoptotic indexes (AI-1 and AI-2) to calculate the apoptotic profile of these lesions. The higher these coefficients are, the more probable an anti-apoptotic profile is expected. In contrast, an index < 1 indicates an increase in *BAX *or *CASP3 *or a decrease in *BCL-2 *mRNA transcription, favoring apoptosis. It has been shown that Bcl-2 protein forms heterodimers with the Bax protein such that Bcl-2-Bax inhibits apoptosis, whereas Bax-Bax homodimers favor it [19]. Tumour growth depends on the balance between proliferation/apoptotic indexes. In the present paper we demonstrated that most of the salivary gland neoplasm samples showed a higher AI-1 and AI-2 when compared to normal salivary glands, suggesting a predominance of anti-apoptotic behavior in neoplastic cells, which in turn contributes to neoplasia growth (Figure 1). This study is the first to demonstrate that salivary gland tumours present an anti-apoptotic transcriptional signature.

It was reported, using the 3'-end DNA labeling method (TUNEL), that in salivary gland neoplasms apoptosis is inversely associated with Bcl-2 expression, but not related to Bax expression [11]. This result was strengthened by another publication using TUNEL method which described an inverse association between apoptosis and the expression of Bcl-2 in adenoid cystic carcinomas [15]. However, in mucoepidermoid carcinomas such association did not exist [13]. In the present paper we have shown an increased *BCL-2 *mRNA transcription in the salivary tumours compared to normal salivary glands in 78% of the samples. If we consider only the pleomorphic adenoma samples, *BCL-2 *overexpression was even higher, corresponding to 88% of these tumours. This result supports the above mentioned paper by Soini and colleagues (1998) [11], which pointed to a very low apoptotic index (0.01%) in pleomorphic adenomas. Our results are in agreement with another study that described Bcl-2 immunopositivity in 33/35 samples of pleomorphic adenomas investigated [14]. Also, all the pleomorphic adenomas exhibited AI-1 and/or AI-2 higher than normal salivary glands (Figure 1). Altogether, the evidence points to Bcl-2 as an important factor in the salivary gland neoplasms pathogenesis and as a possible molecular target in salivary gland tumour treatment in the future.

We did not analyze other benign/malignant lesions in separate groups, because as they are rather unusual (eg. adenoid cystic carcinoma, cystadenocarcinoma, mucinous cystadenoma) we had only a few fresh samples included in the study. However, the expression profile of the two mucoepidermoid samples included in the study was unique, as one of them revealed an apoptotic tendency (#27) and in previous immunohistochemistry based publications, these lesions did not reveal a high percentage of Bcl-2 positive samples [11,13].

We demonstrated an overall *BCL-2 *mRNA overexpression, as well as an increased AI-1 and AI-2 in the salivary tumours when compared to normal salivary glands, and in addition we found an association between increased tumour size and high cellular proliferation index. Although the malignant and benign group of samples did not show difference in the AI-1/AI-2, the malignant samples showed a statistical significant higher cellular proliferation index. Taking these findings together, it seems that while both, benign and malignant tumours, tend to evade apoptosis, the malignant samples in addition tend to have a higher cell proliferation activity, guaranteeing a growth advantage.

p53 positive samples showed higher *BCL-2 *transcription levels than the negative ones, indicating an association of p53 immunopositivity with an increased AI-1 and a predominantly anti-apoptotic profile. It has been shown that p53 induces apoptosis by repressing the transcription of the anti-apoptotic gene *BCL-2 *and activating the transcription of the apoptotic *BAX *[20]. Such apoptotic function could be inactivated by p53 mutations. The mutated p53 is usually more stable than the wild-type leading to higher levels of p53 and to immunohistochemical detection of such protein. Therefore, p53 immunoexpression in the samples analyzed may reflect loss of apoptosis induction promoted by this protein, which may explain the increased anti-apoptotic activity found in these tumours [21]. According to the last publication of the World Health Organization on Head and Neck tumours, the role of p53 in salivary gland neoplasms is an issue of controversy [1]. In the present paper, we demonstrated an association between malignancy and high proliferation index and between proliferation and p53 positivity. This evidence suggests that p53 (clone DO7) positivity may be used as a marker in salivary tumours as it is associated not only with higher proliferation index but also with an anti-apoptotic profile, both contributing to tumour growth. This apparent importance of p53 staining as a potentially useful marker in salivary neoplasms empowers other findings of association between higher salivary p53 expression in salivary gland tumours and poor survival [17].

## Conclusions

In conclusion, we demonstrate an overall anti-apoptotic transcriptional signature in salivary gland neoplasms and an association of it with p53 immunoexpression. In addition, the higher proliferative activity found in the malignant tumours suggests cell proliferation is advantageous to growth of malignant salivary tumours. We further demonstrate that tumour size is associated with cell proliferation, but not with the transcription of apoptotic genes.

## Competing interests

The authors declare that they have no competing interests.

## Authors' contributions

CCG participated in the design of the study, carried out the molecular studies and the immunohistochemistry reactions and drafted the manuscript. MGD participated in the molecular studies and data acquisition. VFB participated in the immunohistochemistry evaluation and reviewed the manuscript. LDM participated in the design of the study and helped to draft the manuscript. RSG conceived the study, reviewed the diagnosis, evaluated the immunohistochemistry results and performed the statistical analysis. All authors read and approved the final manuscript.

## Pre-publication history

The pre-publication history for this paper can be accessed here:

http://www.biomedcentral.com/1471-2407/12/61/prepub
